# Supplementary material for: New Finnlakevirus Isolate FLiP‐2 Provides Insight Into the Ecology of ssDNA Phages in Flavobacterium Hosts
Source: Environ Microbiol. 2026 Jun 15;28(6):e70346. doi: 10.1111/1462-2920.70346 (PMC13269007; doi:10.1111/1462-2920.70346)
Supplement: Supplementary file 1 — Table S1: Water samples collected from freshwaters in Finland and filtered through 0.2 μm filters. PCR analysis of the DNA extracted from filter membranes using primers targeting either FLiP major capsid protein (MCP) or replication initiation protein (Rep) genes. All positive results are included, even when the PCR product size diverges from respective product size of FLiP. Figure S1: EM image of MaF61 particles. In the upper left corner, a phage particle with an uncontracted tail is visible, while in the lower right, two particles with contracted tails can be observed. Scale bar is 100 nm. Figure S2: Effect of ampicillin (Amp), kanamycin (Kan), tetracycline (Tet) and tobramycin (Tob) to growth of Flavobacterium sp. B330, B167 and B114. Figure S3: Cell morphologies of Flavobacterium sp. B114 grown in Shieh medium in 96 well plates at room temperature, imaged under TEM. (A) B114 cells after 24 h. (B and C) B114 cells after 24 h, grown in presence of FLiP. (D) B114 cells after 24 h in presence of 100 μg ml‐1 ampicillin. (E and F) B114 cells after 24 h in presence of FLiP and 100 μg ml‐1 ampicillin. (G and H) B114 cells incubated in hypoxic condition for 5 days and in normal concentration of oxygen for 3 h. (I) B114 cells incubated in presence of FLiP in hypoxic condition for 5 days and in normal concentration of oxygen for 3 h. Scale bars are 500 nm in A, B, G and I, and 2 μm in C, D, E, F and H. Figure S4: Mean (±SE) cell length (nm) of Flavobacterium sp. B114 measured in Radius Emsis program. 50 cells were measured in each treatment except for B114 hypoxic with FLiP, where only 4 cells were found. A) Cells grown in presence of ampicillin or FLiP or both compared to ampicillin ‐free control. B) Hypoxia treated cells with and without presence of FLiP. Asterisks indicate statistical significance in pairwise comparisons (Dunn's test, * = p < 0.05, ** = p < 0.01) in non‐parametric ANOVA. [file EMI-28-e70346-s002.pdf]

Supporting information file 1

PCR details:

Primer sequences (5'-3'):

FLiP MCP Forward	GAATGTTGTTCGCGGTGCTT
FLiP MCP Reverse	CGACCAATGGGAAGAGGGAG
FLiP Rep Forward	TCAGCGCAAAGGTTAGGCAT
FLiP Rep Reverse	GCTGTGCTAACGCCCAAATC
FLiP genome Forward	GCGCAAAGGTTAGGCATAGC
FLiP genome Reverse	AACTTTACCGCTATCGCCGT

PCR program (MCP and Rep):

95°C	7 min	
95°C	30 s	30x
62°C	30 s	30x
72°C	1 min	30x
72°C	10 min	
12°C	∞	

PCR Program (genome):

98°C	30 s	
98°C	10 s	30x
64.8°C	30 s	30x
72°C	4 min. 36 s.	30x
72°C	10 min.	
12°C	∞	

Table S1. Water samples collected from freshwaters in Finland and filtered through 0.2 µm filters. PCR analysis of the DNA extracted from filter membranes using primers targeting either FLiP major capsid protein (MCP) or replication initiation protein (Rep) genes. All positive results are included, even when the PCR product size diverges from respective product size of FLiP.

**Name of lake or river**	**Municipality**	**Date**	**Positive in PCR**	
			**MCP**	**Rep**
Vuojärvi	Laukaa	26.8.2019	+	-
Vuojärvi	Laukaa	22.8.2022	+	-
Saraavesi	Laukaa	26.8.2019	-	-
Saraavesi	Laukaa	22.8.2022	+	-
Peurunkajärvi	Laukaa	26.8.2019	+	+
Peurunkajärvi	Laukaa	22.8.2022	+	-
Vuonteensalmi	Laukaa	18.9.2019	+	+
Vuonteensalmi	Laukaa	22.8.2022	+	+
Kiesimenjärvi	Saarijärvi	11.8.2019	-	-
Kiesimenjärvi	Saarijärvi	20.8.2022	+	-
Ilveslahti, Likosalmi	Laukaa	22.8.2022	-	-
Haapaniemi, Päijänne	Jyväskylä	22.8.2022	-	-
Tikka, Päijänne	Jyväskylä	20.8.2022	+	+
Äijälänsalmi	Jyväskylä	20.8.2022	+	-
Suuruspää, Jyväsjärvi	Jyväskylä	20.8.2022	+	-
Luonetjärvi	Jyväskylä	21.8.2022	+	-
Kaivovesi	Jyväskylä	22.8.2022	-	-
Tuomiojärvi	Jyväskylä	20.8.2022	+	-
Kuhnamo	Äänekoski	22.8.2022	-	-
Äänejärvi	Äänekoski	22.8.2022	+	-
Mansikkaniemi	Saarijärvi	20.8.2022	-	-
Pielinen	Lieksa	2.8.2019	+	-
Tielampi	Lapinlahti	3.8.2019	+	-
Oulujärvi	Vaala	4.8.2019	+	+
Särkinen	Vaala	4.8.2019	+	-
Pantiolampi	Vaala	4.8.2019	-	-
Haarusjärvi	Kauhava	5.8.2019	+	+
Iiruunjärvi	Alajärvi	6.8.2019	+	-
Kankarisvesi	Jämsä	8.8.2019	+	-
Jämsänjoki	Jämsä	8.8.2019	+	-
Ahvenlampi	Laukaa	16.8.2019	+	-
Elänne	Mänttä-Vilppula	25.8.2019	-	-

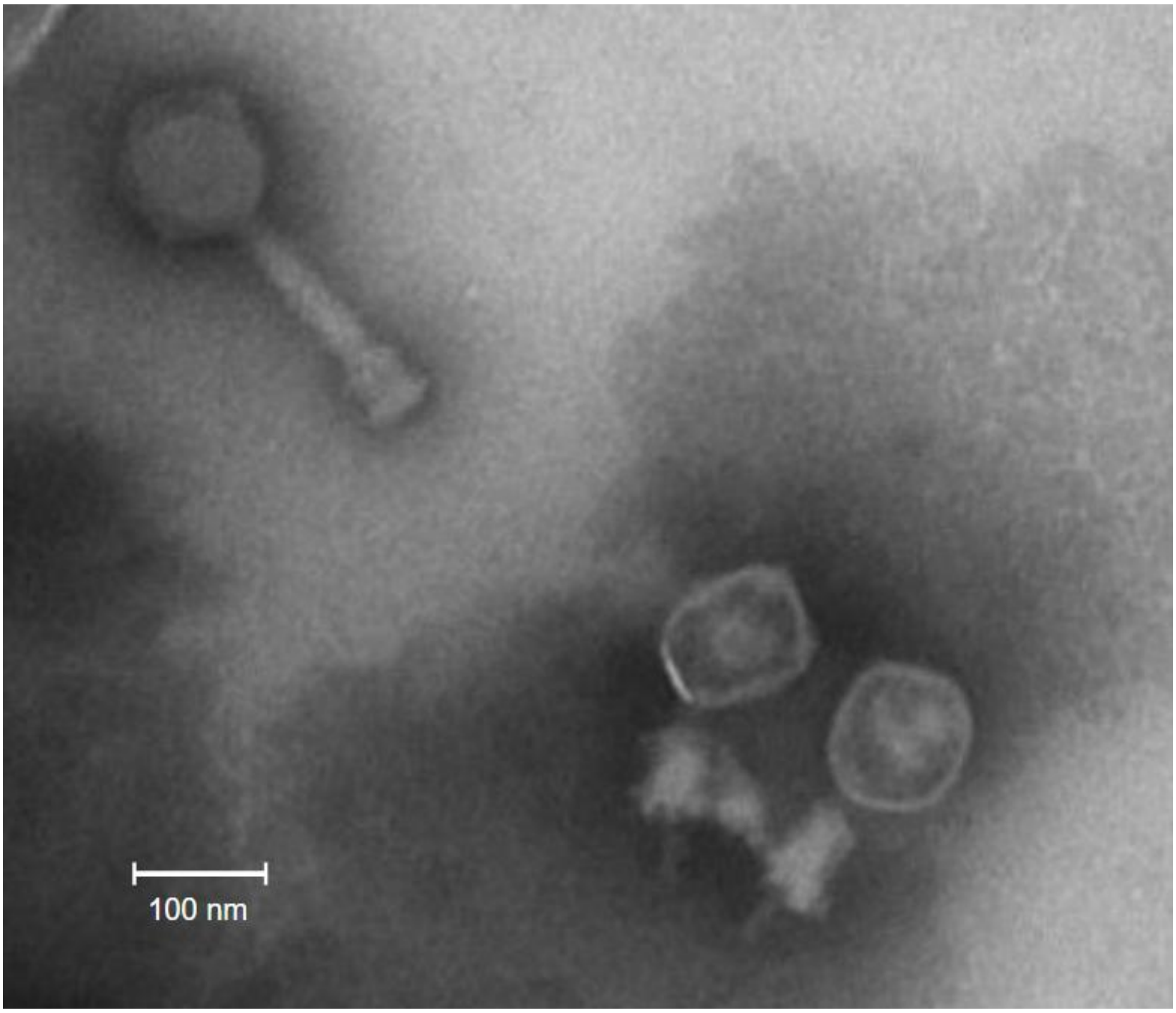

Figure S1. EM image of MaF61 particles. In the upper left corner, a phage particle with an uncontracted tail is visible, while in the lower right, two particles with contracted tails can be observed. Scale bar is 100 nm.

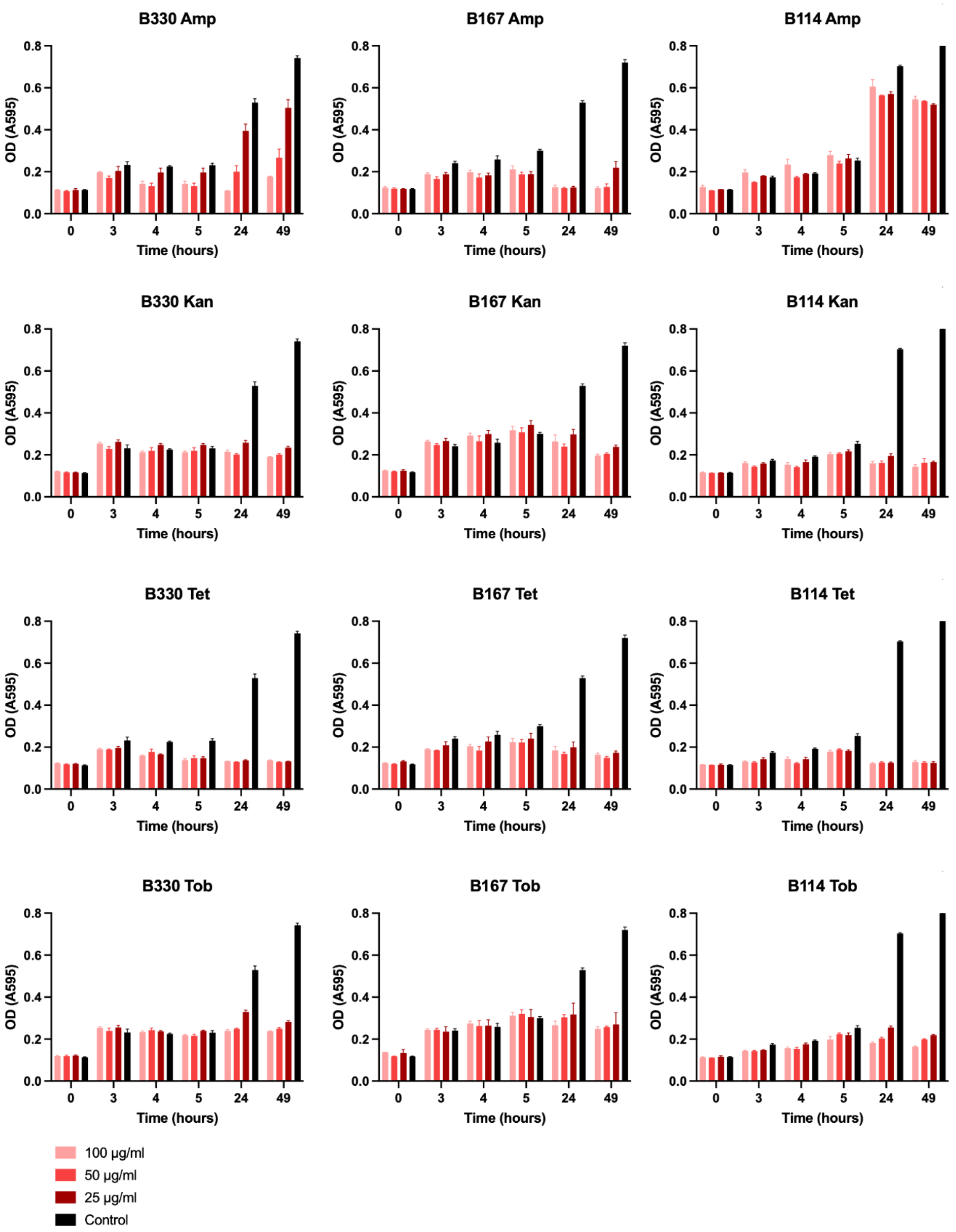

Figure S2. Effect of ampicillin (Amp), kanamycin (Kan), tetracycline (Tet) and tobramycin (Tob) to growth of Flavobacterium sp. B330, B167 and B114.

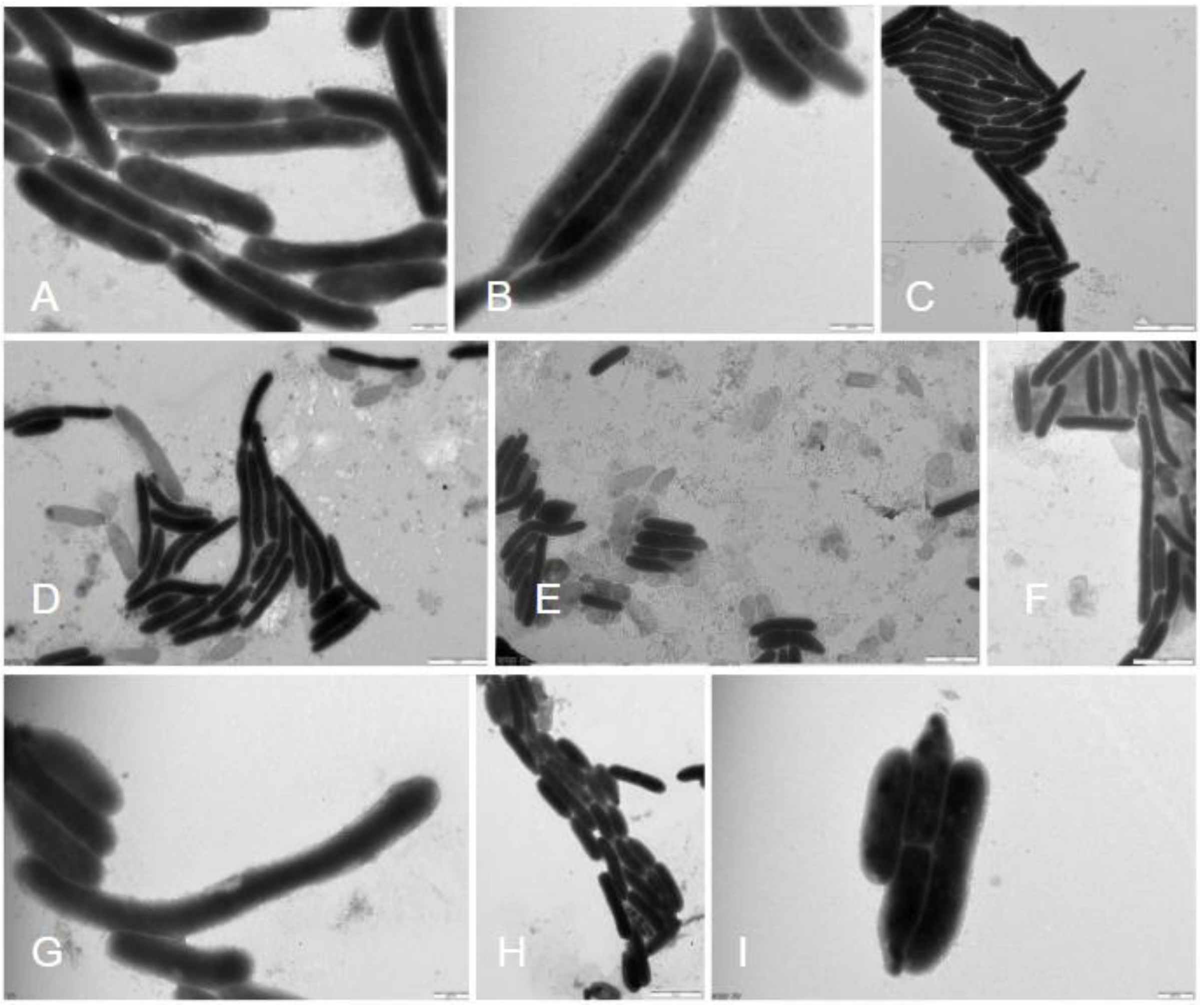

Figure S3. Cell morphologies of Flavobacterium sp. B114 grown in Shieh medium in 96 well plates at room temperature, imaged under TEM. A) B114 cells after 24 hours. B-C) B114 cells after 24 hours, grown in presence of FLiP. D) B114 cells after 24 hours in presence of 100 µg ml^{-1} ampicillin. E-F) B114 cells after 24 hours in presence of FLiP and 100 µg ml^{-1} ampicillin. G-H) B114 cells incubated in hypoxic condition for 5 days and in normal concentration of oxygen for 3 hours. I) B114 cells incubated in presence of FLiP in hypoxic condition for 5 days and in normal concentration of oxygen for 3 hours. Scale bars are 500 nm in A, B, G and I, and 2 µm in C, D, E, F and H.

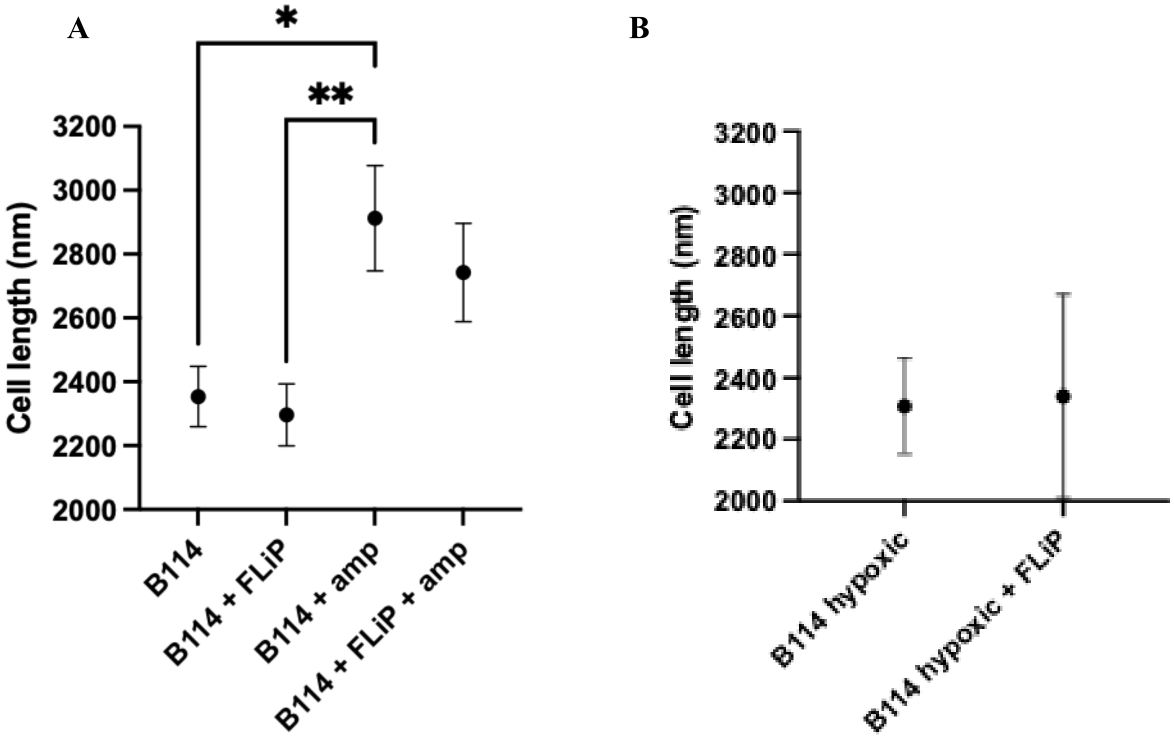

Figure S4. Mean (+/- SE) cell length (nm) of Flavobacterium sp. B114 measured in Radius Emsis program. 50 cells were measured in each treatment except for B114 hypoxic with FLiP, where only 4 cells were found. A) Cells grown in presence of ampicillin or FLiP or both compared to ampicillin-free control. B) Hypoxia treated cells with and without presence of FLiP. Asterisks indicate statistical significance in pairwise comparisons (Dunn's test, * = $p<0.05$, ** = $p< 0.01$) in non-parametric ANOVA.
