# Supplementary material for: New Finnlakevirus Isolate FLiP‐2 Provides Insight Into the Ecology of ssDNA Phages in Flavobacterium Hosts
Source: Environ Microbiol. 2026 Jun 15;28(6):e70346. doi: 10.1111/1462-2920.70346 (PMC13269007; doi:10.1111/1462-2920.70346)
Supplement: Supplementary file 2 — Data S1: Supporting Information. [file EMI-28-e70346-s001.pdf]

ORF name	Start nucleotide	End nucleotide	Nucleotide Length	Direction	Final annotation
ORF1	315	1751	1437	reverse	
ORF2	2026	2301	276	reverse	
ORF3	2294	2677	384	reverse	
ORF4	2677	2889	213	reverse	
ORF5	2897	3757	861	reverse	
ORF6	3934	4176	243	reverse	
ORF7	4193	4492	300	reverse	
ORF8	4496	4789	294	reverse	
ORF9	4797	5054	258	reverse	
ORF10	5065	5343	279	reverse	
ORF11	5345	5608	264	reverse	
ORF12	5608	6369	762	reverse	
ORF13	6436	6630	195	reverse	
ORF14	6633	6935	303	reverse	
ORF15	6925	7179	255	reverse	
ORF16	7182	7718	537	reverse	
ORF17	7708	9531	1824	reverse	
ORF18	9873	10094	222	reverse	
ORF19	10091	10420	330	reverse	
ORF20	10469	10615	147	reverse	
ORF21	10666	10827	162	reverse	
ORF22	11109	11309	201	reverse	
ORF23	11306	11575	270	reverse	
ORF24	11721	11915	195	reverse	
ORF25	11918	12472	555	reverse	
ORF26	12472	12984	513	reverse	
ORF27	13040	13699	660	reverse	
ORF28	13696	14133	438	reverse	
ORF29	14109	14396	288	reverse	
ORF30	14425	14715	291	reverse	
ORF31	14771	15205	435	reverse	
ORF32	15217	15321	105	reverse	
ORF33	15305	15808	504	reverse	
ORF34	15819	16310	492	reverse	
ORF35	16312	16815	504	reverse	
ORF36	16833	17114	282	reverse	
ORF37	17116	17607	492	reverse	
ORF38	17614	17886	273	reverse	
ORF39	18211	18489	279	reverse	
ORF40	18621	18893	273	forward	
ORF41	18883	19143	261	reverse	
ORF42	19275	19544	270	reverse	
ORF43	19576	20238	663	reverse	
ORF44	20235	20450	216	reverse	
ORF45	20453	21103	651	reverse	
ORF46	21100	21627	528	reverse	**Putative SLOG family protein**
ORF47	21617	21943	327	reverse	
ORF48	21933	22127	195	reverse	
ORF49	22349	22540	192	reverse	
ORF50	22540	23028	489	reverse	
ORF51	23126	23482	357	reverse	**Putative CHC2 zinc finger containing protein**
ORF52	23482	24684	1203	reverse	
ORF53	24728	24961	234	reverse	
ORF54	24951	27596	2646	reverse	
ORF55	28420	29502	1083	forward	
ORF56	29499	32120	2622	forward	
ORF57	32187	32435	249	forward	
ORF58	32445	34034	1590	forward	**Putative helicase**
ORF59	34031	34768	738	forward	
ORF60	34780	35904	1125	forward	**Puttive nuclease activity**
ORF61	35915	36361	447	forward	
ORF62	36416	37003	588	forward	**Putative nucleotide pyrophosphorylase**

ORF63	36981	37802	822	forward	
ORF64	37807	38370	564	forward	**Putative chaperone**
ORF65	38384	40072	1689	forward	**Putative chaperone**
ORF66	40221	40373	153	forward	
ORF67	40409	43393	2985	forward	**Putative DNA polymerase**
ORF68	43583	44200	618	forward	
ORF69	44233	47292	3060	reverse	**Putative tail sheath**
ORF70	47362	47493	132	reverse	
ORF71	47608	50403	2796	reverse	
ORF72	50535	52541	2007	reverse	**Putative helicase**
ORF73	52704	53246	543	reverse	**Putative dihydrofolate reductase**
ORF74	53331	54722	1392	reverse	**Putative helicase/primase**
ORF75	54739	55476	738	reverse	
ORF76	55593	57365	1773	reverse	
ORF77	57375	58094	720	reverse	
ORF78	58097	58258	162	reverse	**Putative chaperone**
ORF79	58275	59636	1362	reverse	**Putative helicase**
ORF80	59691	60998	1308	reverse	
ORF81	61040	61273	234	forward	
ORF82	61275	61955	681	reverse	
ORF83	62001	62615	615	reverse	**Putative baseplate wedge protein**
ORF84	62619	63272	654	reverse	**Putative DNA end protecting protein**
ORF85	63316	63699	384	forward	
ORF86	63758	64360	603	reverse	
ORF87	64320	64523	204	reverse	
ORF88	64532	64810	279	reverse	
ORF89	64820	65260	441	reverse	
ORF90	65321	65791	471	reverse	
ORF91	65805	66467	663	reverse	
ORF92	66471	67625	1155	reverse	**Putative thymidylate synthetase**
ORF93	67707	68411	705	reverse	
ORF94	68586	69206	621	reverse	
ORF95	69188	70024	837	reverse	**Putative guanylate kinase**
ORF96	70098	71411	1314	reverse	
ORF97	71500	72132	633	forward	**Putative hydrolase**
ORF98	72167	72355	189	forward	
ORF99	72339	72542	204	reverse	
ORF100	72549	72845	297	reverse	
ORF101	72845	73624	780	reverse	**Putative baseplate protein**
ORF102	73699	75603	1905	forward	
ORF103	75645	77216	1572	reverse	**Putative baseplate wedge protein**
ORF104	77246	78010	765	reverse	**Putative endonuclease**
ORF105	78049	80061	2013	forward	
ORF106	80063	83206	3144	forward	
ORF107	83270	85345	2076	forward	
ORF108	85417	86064	648	reverse	
ORF109	86100	86387	288	reverse	
ORF110	86395	86685	291	reverse	
ORF111	86688	86939	252	reverse	
ORF112	86943	87311	369	reverse	
ORF113	87413	87964	552	reverse	
ORF114	87964	88302	339	reverse	
ORF115	88389	91067	2679	forward	
ORF116	91070	91561	492	forward	**Putative head completion protein**
ORF117	91554	91925	372	reverse	
ORF118	91950	93131	1182	reverse	**Putative DNA primase/helicase**
ORF119	93234	94436	1203	reverse	
ORF120	94589	94993	405	reverse	
ORF121	95038	95433	396	reverse	
ORF122	95698	96267	570	reverse	
ORF123	96272	99046	2775	reverse	**Putative nudix hydrolase**
ORF124	99059	99814	756	reverse	
ORF125	99826	100794	969	reverse	

ORF126	100791	101453	663	reverse	
ORF127	102287	102688	402	forward	
ORF128	102636	104255	1620	forward	
ORF129	104308	104769	462	forward	
ORF130	104766	105500	735	forward	
ORF131	105670	105957	288	forward	**Putative DNA binding protein**
ORF132	106029	106430	402	reverse	
ORF133	106442	109003	2562	reverse	
ORF134	109068	109832	765	forward	**Putative neck protein**
ORF135	109839	110087	249	reverse	
ORF136	110089	111072	984	reverse	**Putative sliding clamp protein**
ORF137	111157	111639	483	reverse	**Putative peptidase/plate protein**
ORF138	111742	112755	1014	forward	**Putative ribonucleoside reductase beta**
ORF139	112888	113595	708	forward	
ORF140	113692	117906	4215	forward	**Putative prohead scaffold**
ORF141	118051	119394	1344	forward	**Putative adenylosuccinate synthetase**
ORF142	119418	121289	1872	forward	**Putative terminase large subunit**
ORF143	121438	123144	1707	forward	**Putative ribonucleoside reductase alpha**
ORF144	123173	123388	216	forward	
ORF145	123471	125399	1929	forward	**Putative portal protein**
ORF146	125545	127206	1662	forward	**Putative major capsid protein**
ORF147	127316	127843	528	reverse	**Putative baseplate wedge protein**
ORF148	127955	129646	1692	reverse	
ORF149	129735	131090	1356	forward	**Putative adenylosuccinate lyase**
ORF150	131087	131665	579	reverse	
ORF151	131717	132250	534	forward	
ORF152	132257	133225	969	forward	**Putative endopeptidase**
ORF153	133276	136560	3285	reverse	
ORF154	136761	137570	810	forward	
ORF155	137610	138587	978	forward	
ORF156	138580	138750	171	forward	
ORF157	138798	139484	687	forward	
ORF158	139481	139900	420	reverse	
ORF159	139939	140550	612	reverse	
ORF160	140555	141133	579	reverse	**Putative endonuclease**
ORF161	141133	143094	1962	reverse	**Putative DNA ligase**
ORF162	143181	143567	387	reverse	**Putative endonuclease**
ORF163	143571	144095	525	reverse	**Putative glycoside hydrolase**
ORF164	144189	144629	441	reverse	
ORF165	144633	145253	621	reverse	
ORF166	145241	145546	306	reverse	
ORF167	145284	145433	150	forward	
ORF168	145461	145643	183	forward	
ORF169	145664	146389	726	reverse	
ORF170	146422	147570	1149	reverse	
ORF171	147563	147745	183	reverse	
ORF172	147757	151512	3756	reverse	
ORF173	151538	151645	108	reverse	
ORF174	151666	151965	300	reverse	
ORF175	151969	153396	1428	reverse	**Putative leucine rich repeat protein**
ORF176	153401	155008	1608	reverse	**Putative leucine rich repeat protein**
ORF177	155023	159075	4053	reverse	
ORF178	159186	159737	552	reverse	
ORF179	159766	160479	714	reverse	
ORF180	160470	160751	282	reverse	
ORF181	160820	161338	519	reverse	
ORF182	161494	161748	255	reverse	
ORF183	161748	162197	450	reverse	
ORF184	162172	162672	501	reverse	
ORF185	162706	163071	366	reverse	
ORF186	163077	163442	366	reverse	
ORF187	163461	163820	360	reverse	
ORF188	163829	164506	678	reverse	

ORF189	164503	165036	534	reverse
ORF190	165020	165349	330	reverse
ORF191	165367	165699	333	reverse
ORF192	165701	166381	681	reverse
ORF193	166493	167233	741	reverse
ORF194	167243	167563	321	reverse
ORF195	167562	167711	150	forward
